# Caproic Acid-Producing Bacteria in Chinese Baijiu Brewing

**DOI:** 10.3389/fmicb.2022.883142

**Published:** 2022-05-04

**Authors:** Siqi Yuan, Ziyang Jin, Ayaz Ali, Chengjun Wang, Jun Liu

**Affiliations:** ^1^School of Biological Engineering, Sichuan University of Science & Engineering, Zigong, China; ^2^Luzhou Laojiao Group Co. Ltd., Luzhou, China; ^3^Key Laboratory of Brewing Biotechnology and Application of Sichuan Province, Yibin, China; ^4^Wuliangye Group Co. Ltd., Yibin, China

**Keywords:** n-caproic acid, Chinese Baijiu, synthetic pathway, *Clostridium kluyveri*, caproic acid-producing bacteria

## Abstract

Caproic acid can be used as spices, preservatives, animal feed additives, and biofuels. At the same time, caproic acid plays an important role in Chinese Baijiu. It is the precursor substance for the synthesis of ethyl caproate, which directly affects the quality of Chinese Baijiu. Caproic acid-producing bacteria are the main microorganisms that synthesize caproic acid in Chinese Baijiu, and the most common strain is *Clostridium kluyveri*. Caproic acid-producing bacteria synthesize n-caproic acid through reverse β-oxidation to extend the carboxylic acid chain. This method mainly uses ethanol and lactic acid as substrates. Ethanol and lactic acid are converted into acetyl-CoA, and acetyl-CoA undergoes a series of condensation, dehydrogenation, dehydration, and reduction to extend the carboxylic acid chain. This review addresses the important issues of caproic acid-producing bacteria in the brewing process of Baijiu: the common caproic acid-producing bacteria that have been reported metabolic pathways, factors affecting acid production, biological competition pathways, and the effect of mixed bacteria fermentation on acid production. It is hoped that this will provide new ideas for the study of caproic acid-producing bacteria in Chinese Baijiu.

## Introduction

Chinese Baijiu is a distilled spirit made from grains, and it has a high reputation and constitutes an important part of Chinese dietary profile. Chinese Baijiu has a long history of more than 2,000 years, and it is one of the six major distilled spirits in the world. The main components of Baijiu are ethanol and water, which account for about 98%, and the trace components acids, esters, and alcohol that comprise the remaining 2–3% (Chai et al., 2021). Based on the difference in the temperature used to make koji (a starter of Chinese Baijiu Brewing) and the difference in the production process, Baijiu can be divided into strong-flavor, sauce-flavor, Fen-flavor, Phoenix-flavor, and so on (Fang et al., 2018). Strong-flavor Baijiu is the most popular, and its output accounts for 70% of the total output of Chinese baijiu (Zou et al., 2018).

In the process of Baijiu brewing, caproic acid is used as a precursor for the synthesis of ethyl caproate. Caproic acid is a linear, saturated fatty acid that contains six carbons with a molecular formula of C_6_H_12_O_2_. It is an oily colorless or yellow liquid with a pungent smell, slightly soluble in water and soluble in ethanol. Caproic acid is used widely in spices, preservatives, and antibacterial agents. In the food industry, caproic acid itself is an edible flavor and can be used as a food additive in butter and bread. In the pharmaceutical industry, a variety of pharmaceutical products can be synthesized from caproic acid as a source of ethyl, such as zinc acetate caproate for the treatment of gastric ulcers and aminocaproic acid tablets for hemostasis. In livestock breeding, caproic acid can be used as an additive in animal feed (Van Immerseel et al., 2004), which has great development potential as an antibiotic substitution. At the same time, it is also a natural antibacterial agent, which improves intestinal microbial flora and enhances animal immunity (Desbois, 2012). In addition, although caproic acid itself cannot be used directly as a fuel, it can be used as a precursor to produce liquid fuel.

Caproic acid is being used more widely, and the source of caproic acid is becoming more important. There are three main methods to produce caproic acid. The first is to hydrolyze and to separate this acid from natural coconut oil or palm oil, but this method is limited by raw materials, and the extraction is very inefficient. The second is the chemical synthesis method. At present, the main synthetic method for producing caproic acid is to drop nitric acid into 2-octanol and to oxidize it in the presence of a catalyst to obtain caproic acid. The third is microbial fermentation, which uses caproic acid-producing bacteria to convert raw materials into caproic acid through specific microbial metabolic pathways (Vasudevan et al., 2014).

Caproic acid-producing bacteria are microorganisms that can produce caproic acid by microbial fermentation with ethanol, acetic acid, glucose, D-galactitol, and lactic acid as carbon sources (Yang et al., 2020). In the process of Baijiu fermentation, caproic acid-producing bacteria can synthesize ethyl caproate by using caproic acid and ethanol through esterification reaction. The composition of strong-flavor Baijiu is determined by the diversity of microorganisms in the pit mud. Strong-flavor Baijiu contains more than 1,300 trace components, of which ethyl caproate is the main aroma (Tao et al., 2014). The concentration of ethyl caproate affects the quality of strong-flavor Baijiu, so caproic acid-producing bacteria are the main functional bacteria in the pit when brewing strong-flavor Baijiu. In sauce-flavor Baijiu, ethyl caproate is not used as an indicator. A high concentration of ethyl caproate in sauce-flavor Baijiu causes the flavor profile to be biased. Therefore, the growth of caproic acid-producing bacteria is restricted during the fermentation process. Fen-flavor Baijiu is affected by the brewing process. It is fermented in an underground tank, and it is strictly sterilized during fermentation to prevent the growth of caproic acid-producing bacteria. The concentration of ethyl caproate in Fen-flavor Baijiu is also very low, and the composition of ethyl caproate has little relationship with caproic acid-producing bacteria. Phoenix-flavor Baijiu contains an aroma due to a combination of ethyl acetate and ethyl caproate. Although mud tanks are also used for fermentation, new pit mud is replaced every year to strictly control the growth of caproic acid-producing bacteria. Therefore, caproic acid-producing bacteria play a vital role in the process of Baijiu brewing. Breeding high-producing caproic acid strains and optimizing the conditions for caproic acid production by caproic acid-producing bacteria are the current “hot” topics in research in the Baijiu industry.

## Common Caproic Acid-Producing Bacteria in Chinese Baijiu Brewing

### Clostridium kluyveri

At present, most of the caproic acid-producing bacteria that are used are obligate anaerobic bacteria in the genus *Clostridium*, and the most widely used is *Clostridium kluyveri* (Table 1). It exists widely in pit mud, river bottom sludge, and rumen of cattle. It synthesizes caproic acid with ethanol, propanol, acetic acid, butyric acid, and succinic acid as substrates, but it cannot use glucose or other sugars (Spirito et al., 2014). *Clostridium kluyveri* is regarded as the model strain of caproic acid-producing bacteria. The first caproic acid bacterium *C. kluyveri* was isolated from the mud from the Delft canal in the Netherlands by Barker in 1937. The strain synthesized butyric acid and caproic acid with ethanol and acetic acid as substrates, and it produced trace butanol and hexanol at the same time (Barker and Taha, 1942). Subsequently, Bornstein and Barker (1948) found that when the ethanol content in the substrate was too high, the yield of caproic acid was higher than butyric acid. The product *C. kluyveri* depended on the concentration and proportion of the substrate, which was acetic acid and ethanol (Bornstein and Barker, 1948). Weimer and Stevenson (2012) isolated *C. kluyveri* from the rumen of cattle, and they found that when the molar ratio of ethanol to acetic acid was 5.8:1, the production of caproic acid reached its highest level. Yin et al. (2017) optimized the different molar ratios of ethanol and acetic acid and found that when the ethanol/acetic acid ratio was 10:1, the production of caproic acid reached its highest concentration of 8.42 g L^−1^. These results paved the way for the biosynthesis of caproic acid using industrial fermentation of wastewater (Yin et al., 2017).

### Ruminococcaceae

*Ruminococcaceae* is a Gram-positive, anaerobic bacterium, which exists widely in the rumen of cattle and sheep. Zhu et al. (2017) isolated a rumen bacterium that produced caproic acid from the mixed bacteria in pit mud and named it CPB6 (Table 1). The strain used lactic acid as a carbon source and organic wastewater that contained lactic acid as the substrate to produce caproic acid by continuous fermentation. The yield of caproic acid reached 16.60 g L^−1^, compared with microorganisms that were reported previously that used ethanol as a substrate, and the ability to produce caproic acid was improved significantly (Zhu et al., 2017). CPB6 cannot use ethanol to synthesize acetyl-CoA to produce caproic acid due to the deletion of the gene that encodes acetaldehyde dehydrogenase, and there are great differences in the gene that encodes the key enzyme for caproic acid synthesis compared with *C. kluyveri*.

### Megasphaera elsdenii

*Megasphaera elsdenii* is a Gram-negative bacterium, which is strictly anaerobic, and mainly exists in sheep and bovine rumens. It uses carbohydrates, such as lactic acid, glucose, fructose, and sucrose (Kim et al., 2020). Although *M. elsdenii* utilizes more substrates than *C. kluyveri*, the metabolic process is subject to many limitations. For example, when glucose is used as substrate, it produces mainly butyric acid, and the yield of caproic acid is low (Marounek et al., 1989). However, when sucrose, acetic acid, and butyric acid were used as substrates at the same time, the yield of caproic acid increased. Jeon et al. (2016) isolated anaerobic bacteria that produced caproic acid from bovine rumen samples, and the bacterium with the highest caproic acid production was identified as *Megasphaera* sp. MH. MH produced C2–C8 fatty acids with fructose as a substrate, which included caproic acid (Jeon et al., 2016), and it had a strong ability to extend the carbon chain.

### Caproiciproducens galactitolivorans

*Caproiciproducens galactitolivorans* is a Gram-positive bacterium, which is strictly anaerobic (Table 1). Jeon et al. (2010) isolated a new type of *Clostridium* which can produce caproic acid and named it BS-1. BS-1 used D-galactitol as a carbon source to produce ethanol, acetic acid, butyric acid, and caproic acid (Jeon et al., 2010). Later, the comparison of strain BS-1 with other closely related strains showed that strain BS-1 represented a new genus of *Clostridium* Group IV, so it was named *C. galactitolivorans*. Table 1 lists some reported caproic acid producing bacteria and their acid production.

**Table 1:** Types of caproic acid-producing bacteria and acid production.

Strain name	Source	Substrate	Acid production g L^−1^	References
*Clostridium kluyveri* N6	Pit mud	Ethanol, acetic acid	3.05 ± 0.29	Hu et al., 2015
*Clostridium kluyveri* 3231B	Rumen of cattle	Ethanol	12.8	Weimer and Stevenson, 2012
*Clostridium sporosphaeroides* BS-1	Mud	Galactitol	32	Jeon et al., 2013
*Caproiciproducens galactitolivorans* BS-1T	Anaerobic digestion reactor	Galactitol	0.69	Kim et al., 2015
*Eubacterium pyruvativorans*	Sheep rumen	Lactic acid	1.16	Wallace et al., 2004
*Megasphaera elsdenii* NCIMB 702410	Rumen of cattle	Sucrose	6.0	Choi et al., 2013
*Megasphaera elsdenii* MHT	Rumen of cattle	Fructose	5.5–7.5	Jeon et al., 2017
*Caproiciproducens* EA1(T)	Biogas reactor	Fructose	–	Flaiz et al., 2020
*Ruminococcaceae* CPB6	Pit mud	Lactic acid	16.6	Zhu et al., 2017

## The Way to Synthesize Caproic Acid in Chinese Baijiu Brewing Process

**Figure 1:**
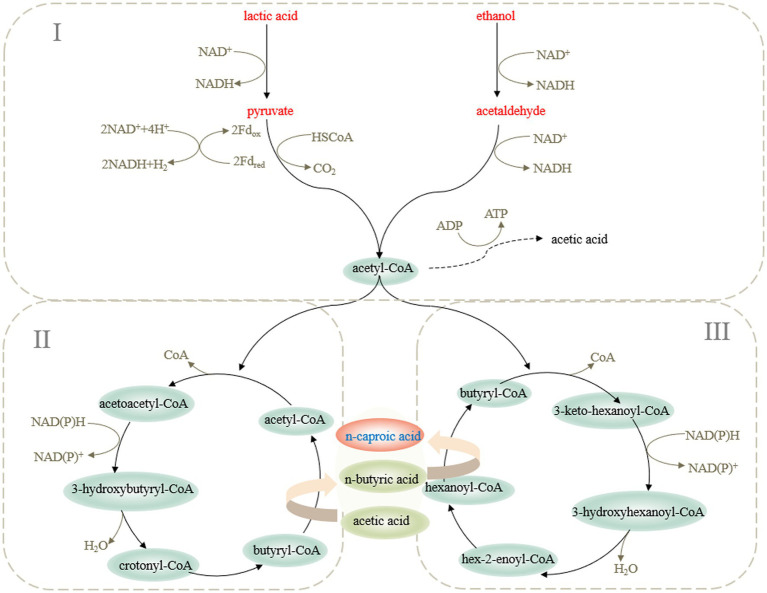
Metabolic pathway of caproic acid production by microorganisms.

The microbial synthesis of medium-chain fatty acids relies on reverse β-oxidation, which uses a small quantity of carbonic acid as a substrate to condense with acetyl-CoA to form β-carbonyl fatty acyl-CoA, and then, it extends the carboxylic acid chain through dehydrogenation, dehydration, and reduction (Vick et al., 2015). In the process of reverse β-oxidation to extend the carboxylic acid chain, ethanol and lactic acid were converted easily into acetyl-CoA to produce NADH (Spirito et al., 2014). NADH provided H ^+^ for the synthesis of caproic acid and promoted the electron flow of the caproic acid metabolic system. Therefore, ethanol and lactic acid were the most ideal electron receptors for the synthesis of caproic acid (Cavalcante et al., 2017). However, the process of converting lactic acid into acetyl-CoA metabolized propionic acid through the acrylate pathway, which resulted in shunting of carbon atoms (Prabhu et al., 2012; Figure 1).

The first step was to synthesize acetyl-CoA. *Clostridium kluyveri* lacks a glycolytic enzyme system and cannot generate pyruvate from glucose through the glycolytic pathway. When it used ethanol and acetic acid as substrates to produce caproic acid, ethanol was first oxidized by alcohol dehydrogenase to produce acetaldehyde, and then, under the action of acetaldehyde dehydrogenase, it produced acetyl-CoA, generated energy, and then it entered the circulation (Seedorf et al., 2008). *Ruminococcaceae* CPB6 and other microorganisms that used substrates, such as glucose or lactic acid, converted the substrate into pyruvate, used pyruvate as an intermediate to generate acetyl-CoA (Zhu et al., 2015), and then underwent reverse β-oxidation to extend the carbon chain.

The second step was to synthesize butyryl-CoA. Two acetyl-CoA were condensed into acetoacetyl-CoA, dehydrogenated to produce 3-hydroxybutyryl-CoA, and dehydrated under the catalysis of 3-hydroxyacyl-CoA dehydratase to produce butenoyl-CoA. Then, butyryl-CoA was obtained by an acyl-CoA reductase reduction. At the same time, under the action of acyl-CoA transferase, acetic acid reacted with butyryl-CoA to generate new acetyl-CoA and butyric acid. At this point, the first carboxylic acid chain extension was completed.

The last step was the synthesis of caproic acid. Butyryl-CoA and acetyl-CoA were converted into hexanoyl-CoA (Kucek et al., 2016). Under the action of acyl-CoA transferase, hexanoyl-CoA reacted with butyrate to produce caproic acid. Zhu et al. (2017) found that when hexanoyl-CoA was sufficient, adding butyric acid and acetic acid used *Ruminococcaceae* CPB6 to produce caproic acid, which indicated that hexanoyl-CoA and acetic acid reacted to produce caproic acid. When producing caproic acid, a part of hexanoyl-CoA participated in the reverse β-oxidation cycle to continue to extend the carboxylic acid chain to produce caprylic acid. However, with the accumulation of caproic acid and other carboxylic acids, the toxicity to cells increased gradually. Because it was limited by energy, it was more difficult to extend the carboxylic acid chain further.

## Effect of Culture Conditions on Production of Caproic Acid

The efficiency of caproic acid synthesis by caproic acid-producing bacteria is closely related to the culture conditions. The main conditions that affect caproic acid production are temperature, pH, and ethanol content. Due to different process conditions, the optimal conditions for caproic acid production are also different. Choosing appropriate conditions is an effective measure to increase the production of caproic acid.

### Temperature

Most caproic acid-producing bacteria are mesophilic and can grow at 30–40°C. For example, *C. kluyveri* can grow at 19–37°C, but the best temperature is 34°C. At present, the mixed bacteria fermentation system for the production of medium-chain carboxylic acid, such as caproic acid, is mostly conducted at 30°C. Agler et al. (2014) found that caproic acid-producing bacteria did not catalyze the synthesis of caproic acid when fermented at 55°C. After gradually reducing the reactor temperature to 30°C, they found that the concentration of caproic acid in the reactor increased rapidly and significantly. This may have been due to the thermodynamic problems of hexanoic acid synthesis that was caused by high temperature, which made the uptake and utilization of electron donors more difficult (Grootscholten et al., 2013).

### pH

The pH of the fermentation environment plays an important role in the growth and acid production of caproic acid-producing bacteria. Neutral conditions are conducive to the synthesis of caproic acid. *Clostridium kluyveri* grows within a pH range of 5.2–8.0, but the optimal pH is 6.8, or close to neutral. As the metabolic process progresses, the concentration of caproic acid in the fermentation environment increases. Caproic acid is not easily soluble in water and accumulates on the surface of cell membranes in the form of molecules, which causes toxicity to cells and affects the absorption of substrates. Ge et al. (2015) found that in a fermentation system with a pH of 5.5, the concentration of caproic acid >7.5 mmol L^−1^ had a significant toxic effect on bacteria. San-Valero et al. (2019) found that *C. kluyveri* produced caproic acid up to 21.2 g L^−1^ when the pH was maintained at 6.8. In addition, pH affected the dissolution state of CO_2_ in the fermentation system, and CO_2_ was a very important inorganic carbon source in the synthesis and metabolism of *C. kluyveri*. With the help of radioisotope research methods, researchers found that 1/3 of the carbon in *C. kluyveri* came from CO_2_ (Jungermann et al., 1968). Therefore, the pH of the fermentation environment was very important for the acidic production of caproic acid-producing bacteria.

### Ethanol Concentration

Ethanol acts as an electron donor in the metabolic pathway in the synthesis of caproic acid. An ethanol concentration that was too low led to the suspension of the reaction during synthesis, and there was not enough substrate to extend the carboxylic acid chain. A concentration that was too high led to a restriction in the growth of caproic acid-producing bacteria and metabolism (Yin et al., 2017). When the ethanol concentration was >40 g L^−1^, the synthesis of caproic acid was inhibited completely (Lonkar et al., 2016). The ratio of ethanol to acetic acid affected the distribution of the product components of the carbon chain extension, which played a key role in the production efficiency of caproic acid (Reddy et al., 2018). The molar ratio of ethanol to acetic acid in the culture system had an important effect on the production of caproic acid. When it was <2:1, all available ethanol in the system reacted with acetic acid to produce butyric acid. When it was >2:1, acetic acid was converted completely into butyric acid, which resulted in a relative excess of ethanol. The excess ethanol continued to promote the synthesis of butyric acid to the caproic acid chain reaction (Spirito et al., 2014).

### Competitive Path

If an efficient fermentation system for producing caproic acid is to be established, the competitive path must be strictly controlled. There are three main competing paths (Table 2): (a) oxidation of acetic acid to produce methane, (b) oxidation of ethanol to acetic acid, and (c) oxidation of medium-chain fatty acids to acetic acid.

**Table 2:** Key reactions of competitive path in the production of caproic acid.

Number	Competitive path	Reaction
1	Oxidation of acetic acid to produce methane	CH_3_COO^−^+H_2_O → CH_4_+HCO^−^
2	Oxidation of ethanol to acetic acid	C_2_H_5_OH+H_2_O → CH_3_COO^−^+H^+^+2H_2_
3	Oxidation of medium-chain fatty acids to acetic acid	C_x_H_2x+1_COO^−^+2H_2_O → C_(x-2)_ H_2(x-2)+1_COO^−^+H^+^+2H_2_

The production of methane is the most common competitive path. Acetic acid-trophic *methanogens* mainly consume the acetic acid substrate to form methane, which leads to the reduction of the electron acceptor for the synthesis of caproic acid. This process formed a competitive relationship with the metabolic pathway to produce caproic acid (Hino and Kuroda, 1993). In the process of synthesizing methane, the increase in CO_2_ decreased the partial pressure of H_2_, so that more ethanol was oxidized to acetic acid and the yield of caproic acid was reduced (Ding et al., 2010). The phenomenon of consuming ethanol through this pathway in the production of caproic acid is called ethanol over-oxidation (Roghair et al., 2018). Medium-chain fatty acids, which included caproic acid, were oxidized to acetic acid under the action of a β-oxidation reaction that caused product loss. The growth of *methanogens* was inhibited by adding bacteriostatic agents and by adjusting the pH (Agler et al., 2014).

In addition to methane bacteria, the growth of acetic acid bacteria produces acetic acid, and the higher acidity of caproic acid lowers the pH of the fermented grains, thereby inhibiting the growth and metabolism of caproic acid-producing bacteria (Liu et al., 2018). Excessive growth of acetic acid bacteria causes an imbalance of trace components in Baijiu. Lactic acid bacteria have a strong reproductive ability. After the fermentation environment is infected with lactic acid bacteria, a large amount of lactic acid is produced. On the one hand, the pH of the environment is reduced. On the other hand, the accumulated lactic acid generates calcium lactate and iron lactate in the pit mud, which inhibits the growth of caproic acid-producing bacteria (Roos and Vuyst, 2017). Therefore, in the brewing process, it is necessary to strictly control the environmental pH, pay attention to the site hygiene, and to prevent the excessive reproduction of acetic acid bacteria and lactic acid bacteria.

## Effect of Co-culture on Caproic Acid-Producing Bacteria

Compared with pure-bred fermentation, mixed bacteria fermentation utilizes the interaction between microorganisms efficiently (Liang and Wan, 2015), makes the growth of microorganisms more stable, and produces higher caproic acid (Table 3).

**Table 3:** Co-culture and acid production of caproic acid-producing bacteria.

Strain name	Substrate	Cultivation method	Acid production	References
*Ruminal microbes* and *C. kluyveri*	Ethanol	Open mixed culture	6.1 g L^−1^	Weimer et al., 2015
*C. autoethanogenum* and *C. kluyveri*	CO, H_2_	Anaerobic mixed fermentation	35 mmol L^−1^	Diender et al., 2016
*Saccharomyces cerevisiae* and *C. klebsiella* 8,022	Ethanol and acetic acid	Anaerobic mixed fermentation	7.09 g L^−1^	Ji et al., 2017
*Megasphaera hexanoica* and *C. tyrobutyricum*	Acetic acid	Open mixed culture	10.08 g L^−1^	Kim et al., 2018
*Clostridium kluyveri* and *Methanogen*	Ethanol	Anaerobic mixed fermentation	–	–
*Caproic acid bacteria* and *Methanogens*	Acetic acid	Binary fermentation	9.85 g L^−1^	Wu, 2007

### Yeast

*Yeast* produces ethanol in the process of metabolism, and *yeast* consumes oxygen during the growth process, which is conducive to the fermentation and the esterification reaction (Shilpa Jain et al., 2016). Co-cultivation of *yeast* and caproic acid-producing bacteria promotes the conversion of synthetic chains to butyric acid, which increases the production of caproic acid (Ge et al., 2015). The esterase produced by *yeast* also esterifies caproic acid to a certain extent, relieves the feedback inhibition effect of excessive caproic acid on caproic acid-producing bacteria, and provides favorable conditions for the synthesis of ethyl caproate. For example, co-cultivation of *Clostridium klebsiella* 8,022 with *Saccharomyces cerevisiae* increased caproic acid production by 12.5% (Ji et al., 2017).

### Clostridium

Among the main caproic acid-producing microorganisms, such as *Clostridium*, there is a case where a metabolite of one *Clostridium* is a substrate of another *Clostridium*. For example, *Clostridium ljungdahlii* assimilates CO_2_ and H_2_ to produce acetic acid and ethanol, which can be used in the fermentation of *C. kluyveri* to produce caproic acid (Richter et al., 2016). Weimer et al. (2015) co-cultured the mixed bacteria in the rumen of dairy cows with *C. kluyveri*, and the production of caproic acid reached 6.1 g L^−1^. Kim et al. (2018) used a fiber membrane bioreactor to ferment with *Clostridium tyrobutyricum* and *Megasphaera hexanoica* in sequence, and the mass concentration of caproic acid was increased to 10.08 g L^−1^.

### Methanogens

There is inter-species hydrogen transfer between caproic acid-producing bacteria and *methanogens* (Tao et al., 2017). The H_2_ and CO_2_ produced by caproic acid-producing bacteria through anaerobic respiration can be used by *methanogens* to produce methane. This reduces hydrogen partial pressure and reduces the free energy of reaction, enables caproic acid-producing bacteria to oxidize ethanol to acetic acid smoothly, and provides favorable conditions for caproic acid-producing bacteria to metabolize carbon compounds thermodynamically (Yan and Dong, 2018). In addition, hydrogen eating methanogens, such as *Methanobacillus omelianskii*, contain methylase system and tetrahydrofolate in cells. When these methanogens die, the released tetrahydrofolate can provide certain growth factors for caproic acid-producing bacteria and promote the proliferation of caproic acid-producing bacteria (Buchenau and Thauer, 2004).

### Actinomycetes

*Actinomycetes* are nitrogen-fixing microorganisms that provide a nitrogen source when co-cultured with caproic acid-producing bacteria. The proteases produced by *actinomycetes* reduce the hydrolysis of proteins into amino acids to provide precursors for the growth and metabolism of caproic acid-producing bacteria. Amylase and cellulase hydrolyze some high molecular substances, such as starch and cellulose, into small molecular substances, and then, they produce low molecular substances, such as ethanol, acetic acid, and ethyl acetate, through the action of bacteria and *yeast* (Guo et al., 2016), which enriches the flavor of Chinese Baijiu. At the same time, the melanin produced by the metabolism of *actinomycetes* has a certain promoting effect on the production of caproic acid (Pompei et al., 2007).

## Application of Caproic Acid-Producing Bacteria in the Baijiu Industry

In the process of Baijiu fermentation, long-term technological operations have enriched the pit mud with microbial flora that are beneficial to winemaking (Zou et al., 2018). They have a wide variety of different functions. Therefore, the pit mud is the basis of the flavor of Baijiu, and the study of co-cultivation using the symbiosis of microorganisms and the metabolic characteristics of microorganisms are important and have applied value. Caproic acid-producing bacteria play a very important function in pit mud, and it has been used widely in Daqu production (Zhang, 2006), grain fermentation (Hu et al., 2018), maintenance of artificial pit mud (Sang et al., 2017), and other-related applications in Baijiu production and research fields. These bacteria inoculate compound functional bacterial liquid or compound intensified bacterial liquid.

For example, spraying caproic acid-producing bacteria liquid and glucoamylase in the mash to produce strong-flavor Baijiu improves Baijiu yield and the physical and chemical indexes effectively. High-quality artificial pit mud can be constructed by adding isolated caproic acid-producing bacteria solution to pit mud and loess and mixing it according to a certain ratio (Liu et al., 2019). Caproic acid-producing bacteria also play an important role in the maintenance of pit mud. The addition of caproic acid-producing bacteria to pit mud increases the number of functional microorganisms in the pit mud significantly and the ethyl caproate concentration in the Baijiu (Du et al., 2019).

In addition, the main aroma component of strong-flavor Baijiu is ethyl caproate. There are two ways to produce ethyl caproate during the brewing process.

- Caproic acid and ethanol react through COA-SH to produce ethyl caproate.
  CH_3_(CH_2_)_4_COOH + ATP + COA-SH →  (CH_3_)_4_CO·SCOA + AMP + Ppi
  CH_3_(CH_2_)_4_CO·SCOA + CH_3_ CH_2_OH → CH_3_(CH_2_)_4_ COOC_2_H_5_ +  COA-SH
- Ester-producing bacteria use ethyl acetate to produce ethyl caproate.
  CH_3_ COOC_2_H_5_ +  CH_3_CH_2_OH → CH_3_(CH_2_)_2_ COOC_2_H_5_ +  H_2_O
  CH_3_(CH_2_)_2_ COOC_2_H_5_ +  CH_3_CH_2_ OH → CH_3_ (CH_2_)_4_ COOC_2_H_5_ +  H_2_O

The symbiotic fermentation of strains with the ability to synthesize ethyl caproate through esterification has more advantages than using pure strains of caproic acid-producing bacteria (Liu et al., 2018). Therefore, the application of compound caproic acid-producing bacteria liquid in Baijiu production has important research significance.

## Discussion

Caproic acid is a precursor for the synthesis of ethyl caproate that plays an important role in the quality of Baijiu, and caproic acid-producing bacteria are also the basic strains for inoculants in pit mud. The main problems in using caproic acid-producing bacteria involve poor screening and low acid production. Therefore, selecting excellent strains of caproic acid-producing bacteria, improving the acid-producing ability of caproic acid-producing bacteria, and applying the compound caproic acid-producing bacteria liquor to the process for making Baijiu are still the focus of the Baijiu industry.

Because caproic acid-producing bacteria are mostly strictly anaerobic bacteria, it is very difficult to screen them, and the cost of cultivation is very high. So far, few pure caproic acid-producing bacteria have been reported. Using mixed cultures is the main approach at present to improve the acid production capacity of caproic acid-producing bacteria. This changes the conditions of fermentation through metabolic activities among different strains, which improves the yield of caproic acid. The organisms that have been studied have a common effect on caproic acid-producing bacteria to promote the production of caproic acid, and these mainly include *yeast, clostridium*, *methanogens*, and *actinomycetes*. At the same time, the use of molecular modification methods to improve the vitality and stability of the key enzymes in the caproic acid metabolism pathway and to screen domesticated high-producing caproic acid strains is also a potential research direction.

In the process of Baijiu brewing, pit mud ages after long-term use, and the number of functional bacteria, such as caproic acid-producing bacteria, is reduced greatly, which seriously affects the quality of Baijiu and the production rate of superior grades. To prevent pit mud from aging, which shortens the maturity time of new pits, pit mud maintenance and cultivation of artificial pit mud are required. Adding good caproic acid-producing bacteria maintains the pit; adding compound caproic acid-producing bacteria solution to maintain pit mud reduces compaction and degradation of the pit mud. Artificial pit mud can be cultivated using different proportions of caproic acid-producing bacteria liquid, yellow clay, peat, and pit skin mud.

In conclusion, several elements are important for high-quality production of Baijiu: (1) screening and breeding new strains of caproic acid, (2) optimizing the culture conditions, (3) studying the promoting effect of mixed culture modes on the acid-producing ability of caproic acid-producing bacteria, (4) analyzing the mechanism of the relationship and the synergy between the metabolic substrates of microorganisms, and (5) developing functional bacteria liquid with a high yield of caproic acid o be used in Baijiu brewing to increase the content of ethyl hexanoate. These factors are of great significance to the Baijiu industry.

## Author Contributions

SY, ZJ, and JL: conceptualization and writing—review and editing. ZJ, AA, CW, and SY: documentation and data curation. ZJ, AA, and JL: writing—original draft preparation. JL: supervision, project administration, and funding acquisition. All authors contributed to the article and approved the submitted version.

## Funding

This research was funded by Industry-University-Research Collaboration project of Wuliangye Co., Ltd. (grant no.: CXY2020ZR02), Key Research and Development Program of Sichuan Province (grant no. 2021YFS0341, 2020YJ0155), Key Program of Key Laboratory of Brewing Biotechnology and Application of Sichuan Province (grant no. NJ2017-01) and Talent introduction Program of SUSE (2017RCL25).

## Conflict of Interest

SY was employed by Luzhou Laojiao Group Co. Ltd. and CW was employed by Wuliangye Group Co. Ltd.

The remaining authors declare that the research was conducted in the absence of any commercial or financial relationships that could be construed as a potential conflict of interest.

## Publisher’s Note

All claims expressed in this article are solely those of the authors and do not necessarily represent those of their affiliated organizations, or those of the publisher, the editors and the reviewers. Any product that may be evaluated in this article, or claim that may be made by its manufacturer, is not guaranteed or endorsed by the publisher.
